# Supplementary figures and images for: Hybrid female sterility due to cohesin protection errors in oocytes
Source: bioRxiv. 2025 Feb 17:2025.02.16.638358. Preprint. [Version 1] doi: 10.1101/2025.02.16.638358 (PMC11870456; doi:10.1101/2025.02.16.638358)

Fig. S1

A

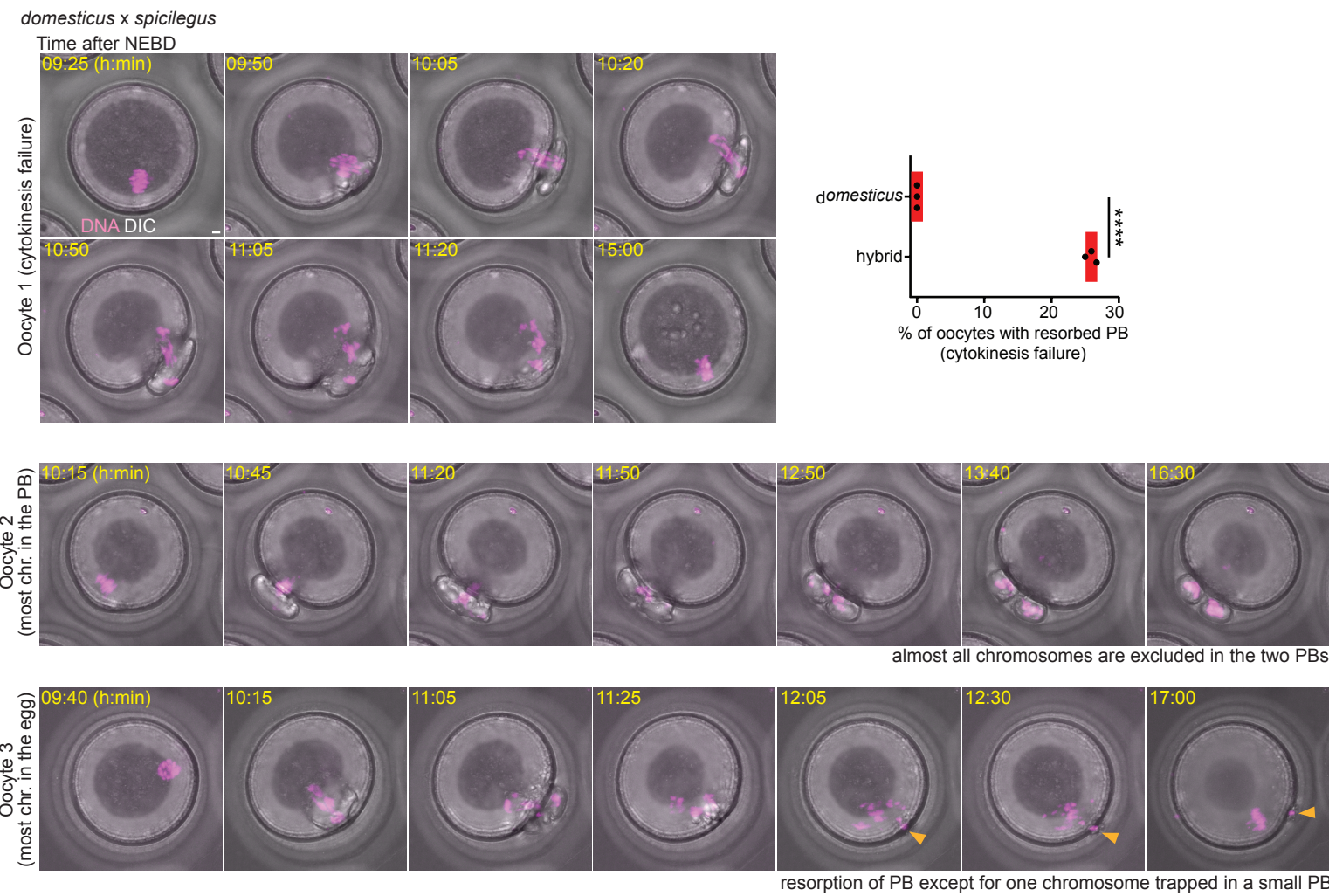

B

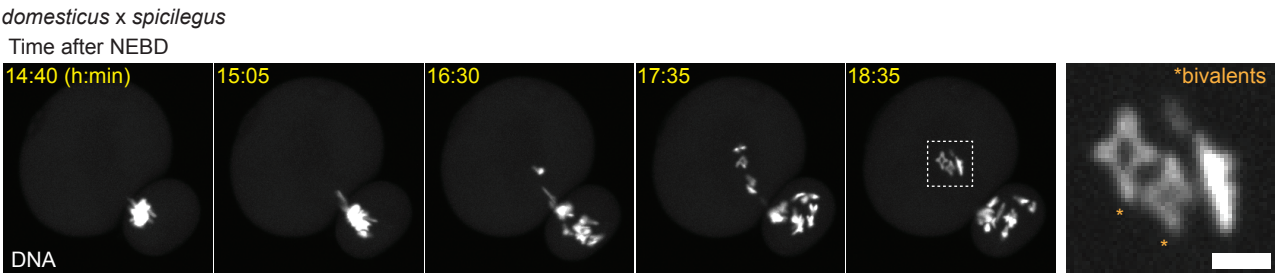

C

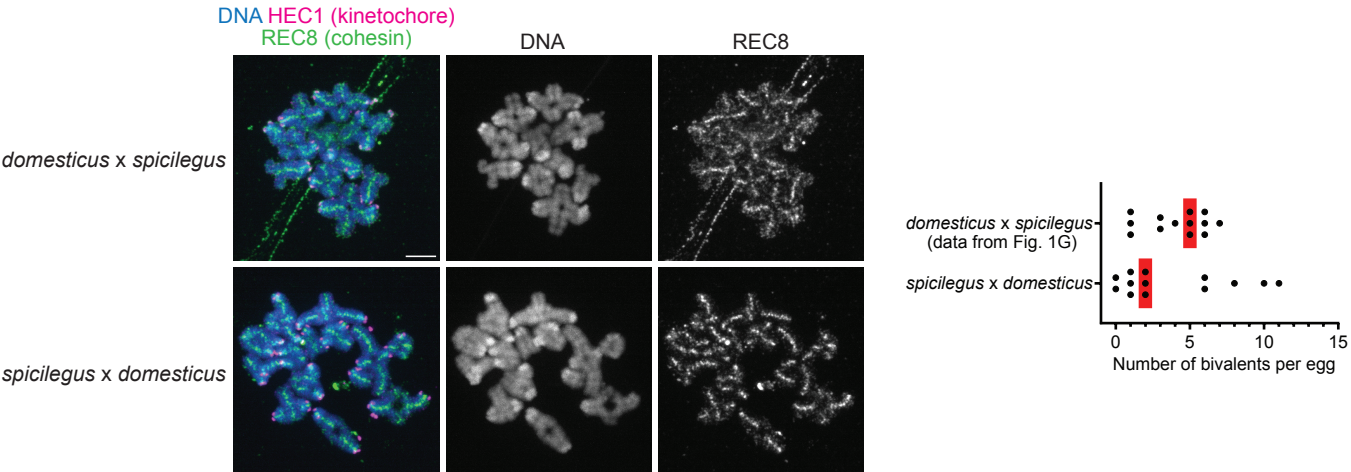

Fig. S2

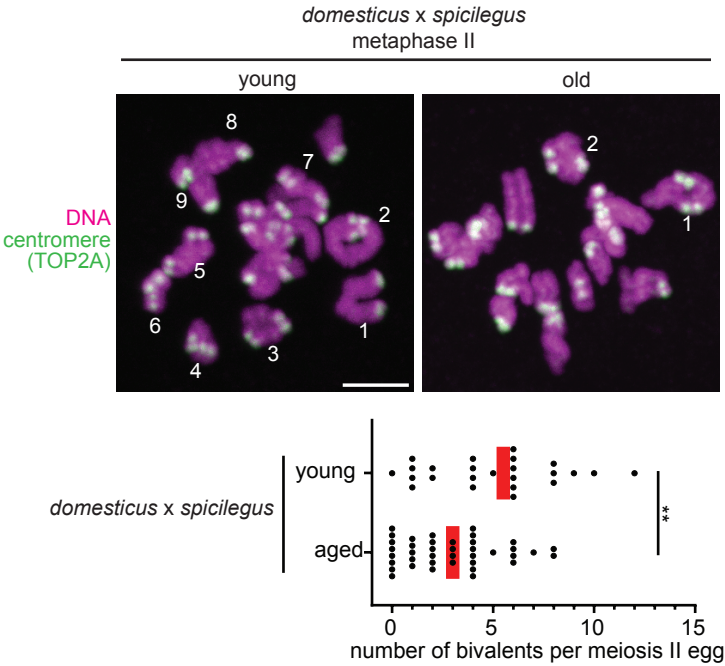

Fig. S3

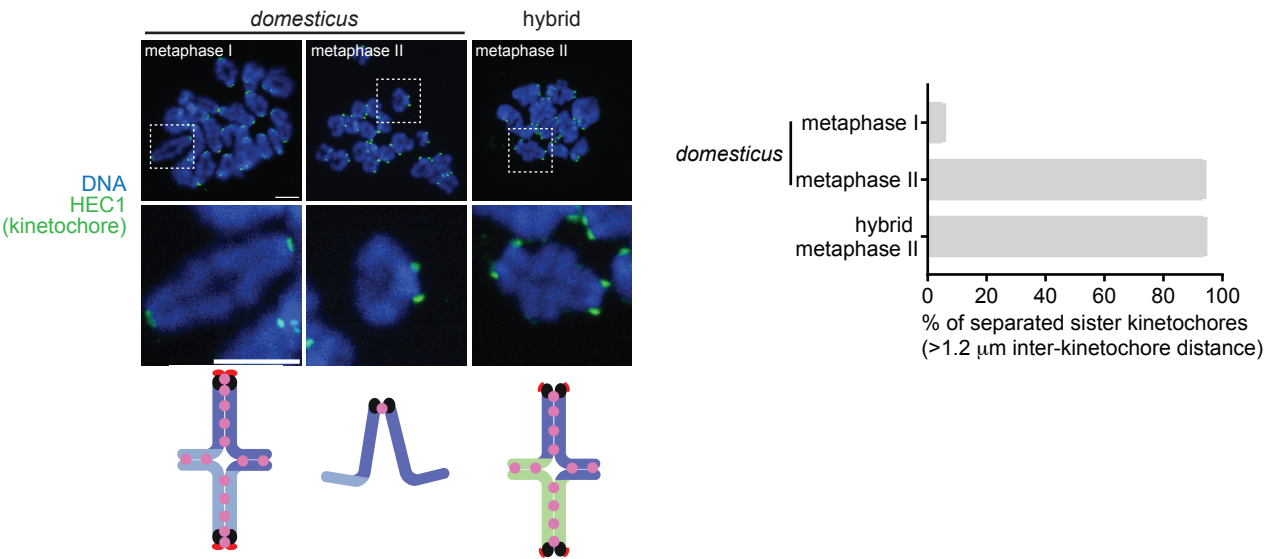

Supplement: Supplement 1 — Fig. S1. Cohesin is maintained along the chromosome arms in hybrid oocytes. (A) Mis-segregation in domesticus x spicilegus oocytes is associated with cytokinetic failures. Images from Fig. 1D were analyzed to quantify the proportion of oocytes with cytokinetic failure. (B) Examples of non-separated bivalent chromosomes captured by live-imaging. The images are from the same time-lapse imaging dataset from Fig. 2A. (C) Chromosome spreads were performed at metaphase II using hybrid oocytes derived from both cross directions (i.e., domesticus x spicilegus and spicilegus x domesticus) and stained for HEC1 and REC8. Graph shows the quantification of the number of bivalents per egg (n = 13 and 13 meiosis II eggs for domesticus x spicilegus and spicilegus x domesticus, respectively); note that the data for domesticus x spicilegus is from Fig. 1G; each dot represents a single egg; red line, mean; unpaired two-tailed t test was used for statistical analysis; ****P <0.0001. Fig. S2. Hybrid meiosis II eggs from older female mice carry less bivalents. Hybrid oocytes from young (3-month old) and aged (12-month old) mice were matured and fixed at metaphase II and stained with TOP2A (centromere). Graph quantifies the number of bivalents in each meiosis II egg (n = 21 and 36 eggs from young ang aged mice, respectively); each dot in the graph represents a single egg; red line, median; Mann-Whitney test was used for statistical analysis; **P <0.01. Fig. S3. Sister kinetochores are normally split in unseparated bivalents. Chromosome spreads were performed at metaphase I (domesticus) and metaphase II (domesticus and the hybrid) and stained for HEC1. Graph shows the quantification of the percentage of chromosomes with separated sister-kinetochores (i.e., inter-kinetochore distance larger than 1.2 μm) (n = 104, 100, and 89 chromosomes for domesticus metaphase I, domesticus metaphase II, and hybrid metaphase II). [file media-1.pdf]
